# Enhancing camera-captured Devanagari documents via geometric filtering for improved vision-language model text extraction

**DOI:** 10.1016/j.mex.2026.103956

**Published:** 2026-05-15

**Authors:** Anup Kelkar, Parag Deshpande, O.G. Kakde

**Affiliations:** a Department of Computer Science and Engineering, Indian Institute of Information Technology (IIIT), Nagpur, Maharashtra, 440024, India; b Department of Computer Science and Engineering, Visvesvaraya National Institute of Technology (VNIT), Nagpur, Maharashtra, 440032, India

**Keywords:** Camera-captured document image, Devanagari character’s geometric properties, Text extraction, VLM platforms

## Abstract

Despite significant advances, modern Vision-Language Model (VLM) platforms remain constrained by the scarcity of training data in non-English languages, limiting their global applicability in Text extraction for regional languages written in Devanagari scripts. Recognizing Devanagari text, particularly in literary materials, poses significant challenges in achieving high accuracy and efficient execution. Effective Text extraction in current VLM platforms must perform several pre-processing steps to retain crucial information while preserving sentence structure and meaning.

• This study proposes an image enhancement method for Devanagari text based on geometric filters and clustering algorithms.

• By leveraging the unique geometrical characteristics of Devanagari characters, the technique accurately detects Devanagari sentences in various font sizes, orientations, and degraded hand-held camera-captured images. It is robust to variations in document rotation, size, and color.

• The method was tested on a dataset of images from books, papers, brochures, and pamphlets, demonstrating superior performance over existing OCR pre-processing techniques for current VLM platforms. The proposed approach achieved a success rate of 95.23 % in locating Devanagari sentences, improving the accuracy of current VLM platforms Text extraction.

## Graphical abstract

**Figure:**
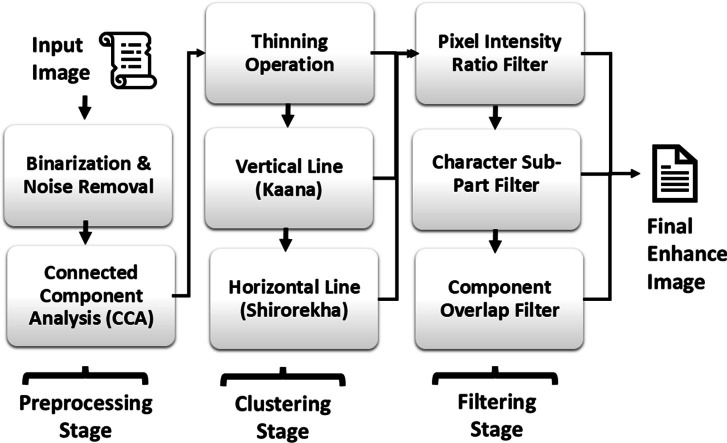

**Specifications table**

**Table:** 

**Subject area**	Computer Science
**More specific subject area**	Computer Vision and Pattern Recognition
**Name of your method**	Geometric Filtering Method for Improved VLM Text Extraction of Devanagari Images.
**Name and reference of original method**	None
**Resource availability**	One of the sample datasets is publicly available on the repository: https://github.com/Shreeshrii/imagesmar/tree/master/printsamples

## Background

India is both a multilingual and a multi-script nation. Indian paperwork is written in 13 different scripts. >520 million people in India use the Devanagari script from the Indo-Aryan family of languages for documentation. The third most-used language worldwide is Hindi, which is also an official language of the Republic of India. Devanagari is the script used for writing Hindi [1]. The ancient Brahmi script is the source of the Devanagari script. Over 118 languages employ the Devanagari writing model that widely embraced worldwide [2]. Many other Indian languages, including Awadhi, Bhojpuri, Chhattisgarhi, Haryanvi, Hindi, Kashmiri, Konkani, Marathi, Nepali, Pali, Rajasthani, Garhwali, Sanskrit, and Sindhi, are written using the same Devanagari script [3].

The Devanagari script, with its unique syntactic and structural traits, presents a complex challenge for current VLM platform Text extraction. These traits include left-to-right writing, a strong tendency toward circular forms that are symmetric inside rectangular edges, and a horizontal line between the two scripts that runs across the leading edge of each character. In Devanagari, this horizontal line is referred to as Shirorekha. The increasing digitization at several Indian government departments has led to a growing interest in identifying Devanagari printed texts. This underscores the urgent need for robust and adaptable preprocessing methods specifically designed for the complexities of the Devanagari script. The current VLM platforms analyse the text from scans or captured images and identifying text, numerals, and objects [4]. There are several uses for script recognition in local languages. However, due to the complex nature of the Devanagari script, which includes compound characters and various types and locations of the modifiers used to generate modified characters, Devanagari Text recognition is a more challenging task than English Script recognition. Several text-line retrieval techniques have been proposed in the past forty years, especially for the English language [4,5]. Most of these algorithms already assume the targeted class's framework, for that is intended, and if they satisfy it, they succeed drastically. For example, the actual output of some open source LLM models suggested in [6] deviates mainly from the ground truth text. No generic filter method, esp. for the Devanagari script language document, can be robustly applied to complex document images [7,8].

This study presents the robust applicability of our filtering approach as a general text-line recognition strategy on a wide range of straightforward and complex document images made up of different greyscale values [9], numerical and non-numerical text [10], different font scripts, as well as other text-line shapes such as in a straight line distorted, and curved text-lines produced by machines that showcase the standard filter approach's effectiveness analysis and benchmark against a variety of data, images in the Devanagari text language [11]. It compares with many popular VLM platforms Text extraction outputs before and after a proposed method application.

## Method details

Now a days, inexpensive mobile devices with cameras are widely accessible and provide flexible, non-contact, and quick document capture. On the one hand, these benefits make mobile cameras a potential scanner replacement for document images. Still, on the other, because of degradation that is not as common in scanned images, the appearance of images acquired by an uncontrolled hand-held camera is inferior to that of scanning the documents. Uneven shading, image blurring, different lighting conditions, character smearing (caused by low resolution), and perspective distortions all contribute to degradation. Such images lose accuracy when directly fed into popular VLM platforms for Text extraction, because of the many distortions in images. The supplied image improvement geometric filter approach that we have suggested applies the filters to such images in the ideal order, immediately improving the input to VLM platforms Text extraction accuracy level. To improve the quality of the input image, we use the Devanagari characters are of similar height, adjacent to each other with half of the height proportion up and down for Matras & Ukaras, usually placed at the identical height across one horizontal line (Shirorekha) of parallel in both dimensions. Also, Devanagari glyphs always have multiple vertical lines as part of themselves or as Kana. Devanagari symbols also typically have comparable distances across them and Kana, along with additional regional parallels. Most of the above characteristics are adhered to by numerous Indian languages that use the script, so the approach can be used to identify and improve the input image to currently popular VLM platforms Text extraction for any language, such as Marathi, Sanskrit, or Konkani.

**Fig. 1:**
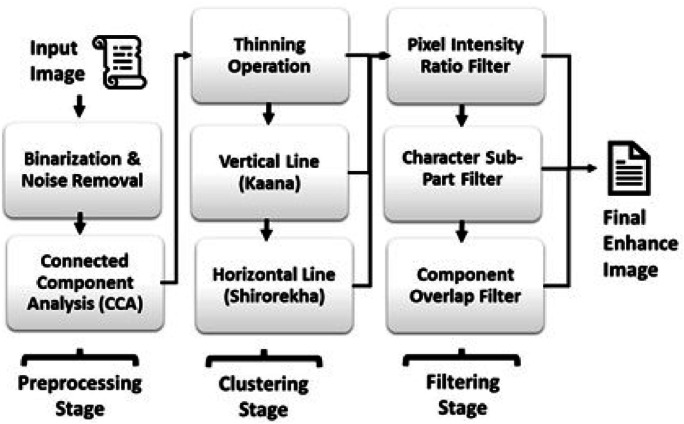
Architecture of proposed model.

The proposed approach as shown in Fig. (1) uses the following steps to create clusters that consists most of the words and characters from the Devanagari script, followed by Filtering methods to retain most of the Devanagari characters.

### Dataset description

**Fig. 2:**
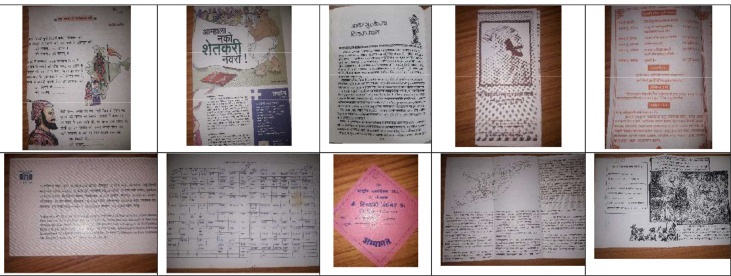
Mobile camera captured sample images.

A public Devanagari dataset available at [12] with ground truth values was utilized with a newly compiled image dataset. The new data set comprises predominantly mobile-captured images encompassing newspaper clippings, brochures, book pages, excel sheets, ID cards, and pamphlets. Newspaper clippings contain 18 % of the database photos, making evaluating how well the algorithms perform in straightforward scenarios more accessible [12]. Most of the image script is in Marathi Devanagari. 11 percent of the images picked contain some graphics, such as the logo, while the remaining 82 percent are plain-text-only images. Only a few solitary characters or one line of text comprises 22 % of the samples. In the database, 82 percent of the images are grayscale, while 18 percent are binary images devoid of colour. Fig. (2) displays a selection of database examples. We will also be making this dataset public soon to utilize and test it with other methods.

### Pre-processing

• Initially, the input document image is converted into grayscale using a threshold and then into a binary format. Uneven lighting and shadows in a source image might make binarization challenging. Bernsen's approach counteracts uneven lighting and shadows, transforming a grayscale image into a binary image. Even when Bernsen's approach is applied, very dark shadows on Devanagari text can result in improper segmentation in the binary image.
• The binary image is now subjected to CCA, and the linked elements are retrieved. Internally, elements are stored in binary form; 1 denotes the ON pixel, and 0 represents the OFF pixel. We identify the geometric characteristics of the components as they are retrieved, including coordinates, width, height, and other dimensions. For smooth working and to collect the different filter algorithms output values, a base table is created, whose column values will be filled as and when the next set of filters are applied.
• The connected components for a text/book page have many small components, sometimes, some significant portion of text may be considered a single component. We will compare the height or width of more significant components and ignore the components whose height or width is more than a configurable percentage value HW_M_, e.g., 50 % of actual image height and width, respectively.
• Components with a pixel height and width below the minimum number of pixels that can be customized M_P_, such as three (3), are ignored as Devanagari text. Lower than that number of pixels for both dimensions would make the characters unrecognizable.

**Fig. 3:**
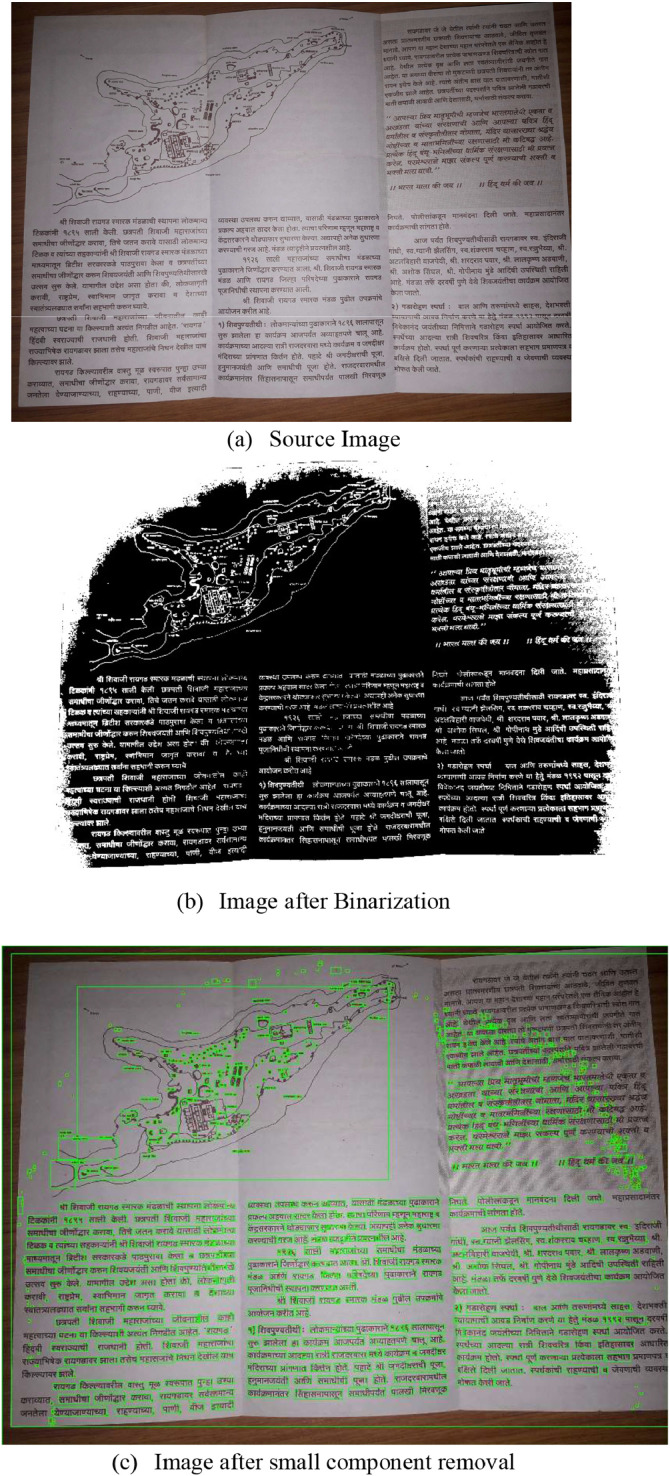
Pre-processing stage.

As a result, after the pre-processing, we established a group of elements with their geometric characteristics, including coordinates, width, height, etc. Fig. (3a) illustrates the source, and Fig. (3b) shows the resultant image after binarization. Fig. (3c) shows the outcomes when similar height/width elements are disregarded, and minor elements are eliminated from the binarized image during pre-processing. It also provides you with the rectangles created on all the connected components, which include the text to be processed by VLM platforms Text extraction and all other removable components.

### Clustering

The pre-processing leftover components are grouped during the clustering stage to identify the potential Devanagari Text components. At this stage, two different clustering operations are carried out sequentially on the remaining components.

**Fig. 4:**
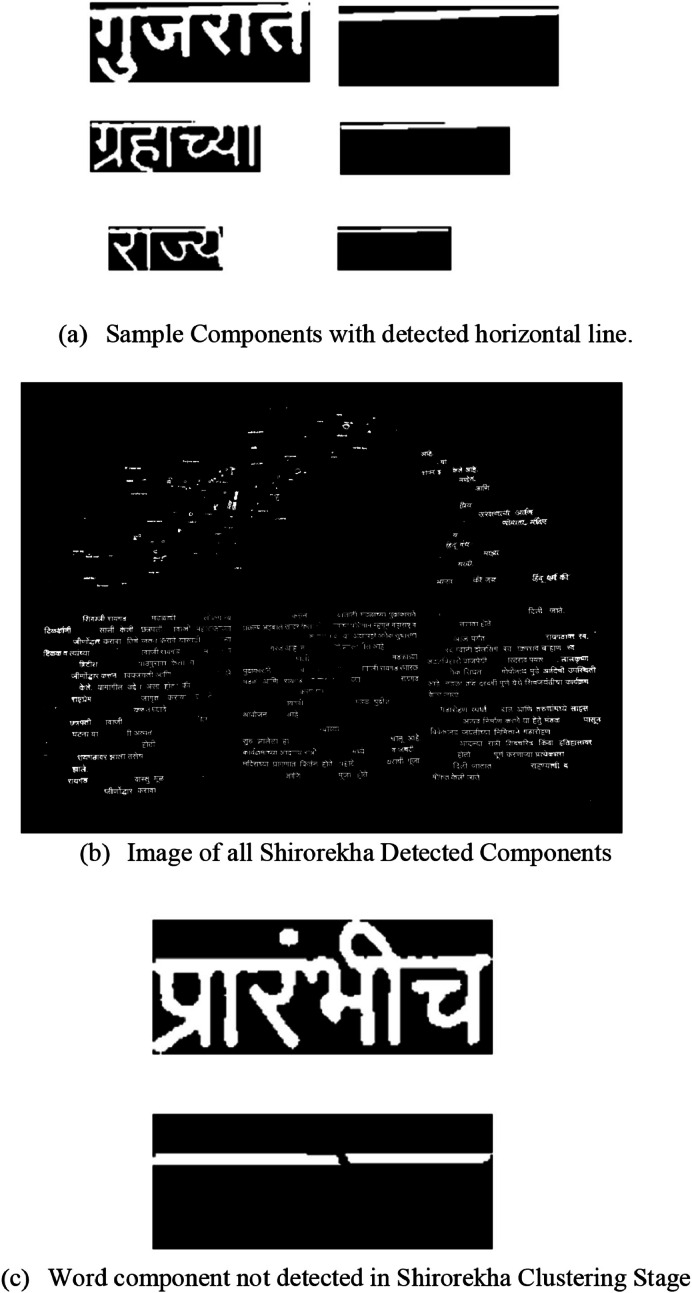
Shirorekha clustering stage.
• Even after detecting maximum Devanagari words using the Shirorekha clustering method, many words still break the Shirorekha in between due to the font style of character representation. Fig. (4c) shows the Devanagari word component example of the same. It has the desired horizontal line but is not continuous; hence, it is not detected in the previous clustering stage on a horizontal line. For such connected components, we utilize another geometrical property of vertical lines, sometimes called “Kana” in the Devanagari context. Advanced morphological transformations of vertical rectangular structuring elements with an Opening operation are utilized to complete the task. Also, we must keep tapping on how many vertical lines can be there in each word, which we initialized as 12 (V_T_).

• Clustering is performed on the horizontal line presence in each word of Devanagari called Shirorekha, and this groups the components into groups that represent the word proportion of the Devanagari text. However, we have used the approval steps in sequence to approve the presence of an actual Devanagari word. Algorithm (1) first uses the Morphology operation to remove the horizontal line from the input components and then make sure the given component has only one horizontal line, not more than that. Secondly, we have fixed a width comparison parameter W_C_ at 65 % of the total width of the component. Also, we need to make sure the line structure is nearly rectangular. If all the conditions are satisfied, we cluster this component as the valid Devanagari word to be present in the final image of any VLM platforms for Text extraction. Fig. (4a) shows sample input components and detected horizontal lines in the same components, and Fig. (4b) shows the actual image of detected components after the horizontal/Shirorekha clustering stage.

**Algorithm 1:** Detect shrirorekha/horizontal line.

**Fig. 5:**
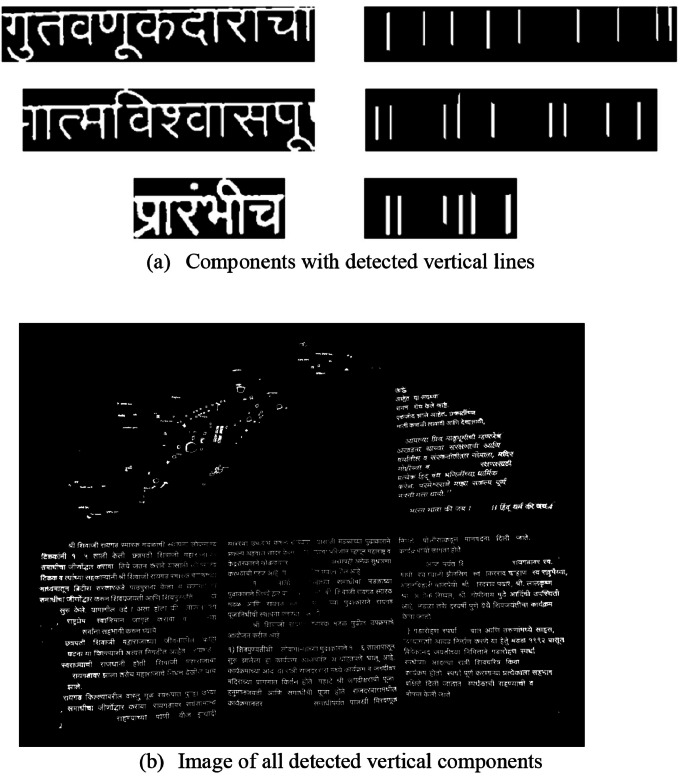
Vertical component clustering stage.

The pseudo-code given in Algorithm (2) starts with the only remaining components after the first Shirorekha Clustering stage and applies the Vertical Morphological Operation. We proceed with only those components that have a maximum of 12 vertical lines and not more than that. After the above step, we checked with all the vertical lines to ensure that the minimum height of all the vertical lines was greater than 50 % of the actual Devanagari component (VH_P_) height. Also, as a final check, detected vertical lines follow the rectangular shapes for confirmation as a part of the Devanagari word. Fig. (5a) shows sample input components and detected vertical lines in the same components, and Fig. (5b) shows the actual image of detected components after the vertical clustering stage. After these Shirorekha and vertical line-based clustering steps are completed, the cluster's components are close to one another and arranged in a straight line, mostly sharing a common Devanagari word. The Devanagari scripts used in many languages exhibit these characteristics. The clustering procedures utilized for the components are comparable to those outlined. After the clustering phases, the identified cluster elements meet all the height- and horizontal-based clustered requirements. However, as shown in Fig. (5b), the final image still does not have many Devanagari word components. The following filtering procedures are used to add these Devanagari word components.

**Algorithm 2:** Detect vertical line (Kana).

### Filtering stage

In the filtering process, each element is meticulously stored as a part of the Devanagari word text, excluding the Shirorekha line and height-based clustering characteristics. The elements that meet the filtering criteria are retained in the rest of the clusters. The filters are determined based on the close relationship of the geometrical characteristics of the Devanagari word characters. This precision in filtering ensures that only the most relevant elements are retained. Below is a detailed description of the filtering procedures employed.

**Fig. 6:**
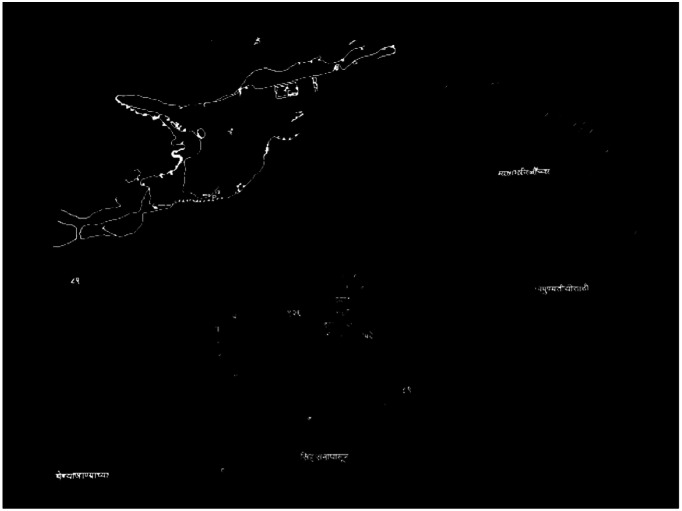
Detection of all thinning components in one image, along with some undesired components.
• Devanagari printed fonts have some drawbacks, especially in many cases of characters that may not be shown as one and may be broken in between, falsely detecting as different components. Also, many times, the numbers shown between the words are missing from the horizontal and vertical filters. We compared their heights with neighbour components for those that maintain their structure after thinning operation. If the element is almost identical, it must be accepted as valid and kept in the final output image for processing. Here in our filter, we've considered the neighbour parameter K_N_=4, which means that the heights of four neighbouring components match those of the current component. The centroid point is the geometric centre of the component, and it is used to determine the surrounding elements for comparison. Fig. (7a) & (b) demonstrate that the actual image is covered with components surrounding already recognized components. If the given component's height matches a neighbouring component's, our algorithm will accept it for further recognition.

• Each element in the rest of the clusters undergoes a thorough inspection, followed by a thinning process. By comparing the binary matrix representations of the components before and after the thinning operation, we ensure that only the most suitable elements are retained. However, the thinning operation keeps parts that have a regular shape but are not Devanagari word parts. Fig. (6) shows the image of detected components after the thinning operation, demonstrating the thoroughness of the inspection process. As can be seen from the thinning filter, the system is designed to adapt to different scenarios. It does not add all the remaining components detected by thinning operations to the original image. Instead, it retains only the elements that satisfy any one of the following filter properties, demonstrating the system's adaptability to different filtering criteria.

**Fig. 7:**
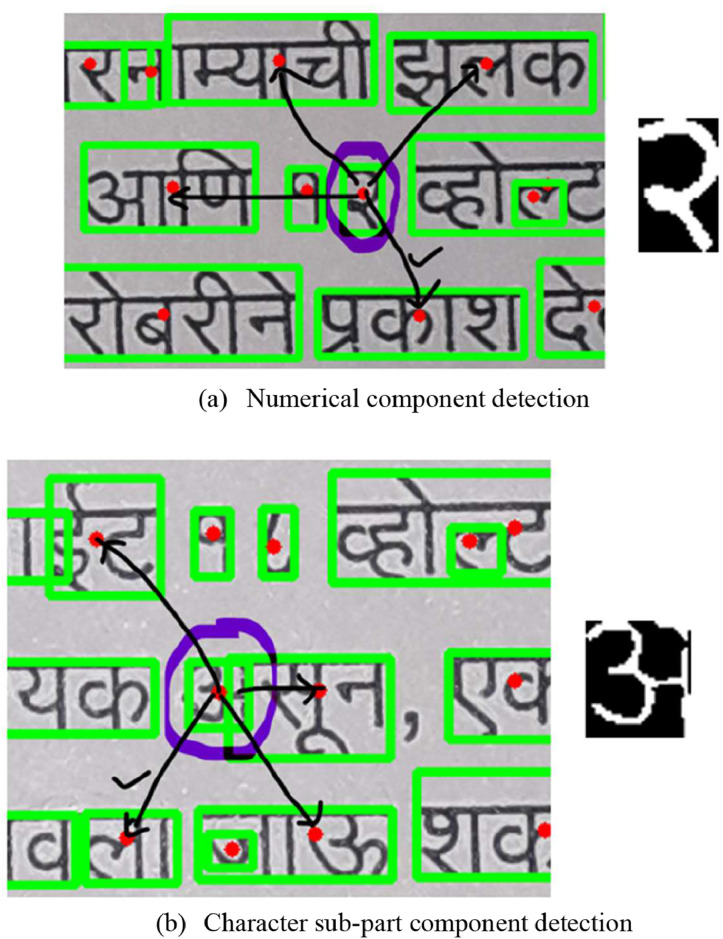
Component detected by equal height filter.
• If the remaining elements are still part of the Devanagari word character, the components discovered by thinning procedures also adhere to a desirable pixel intensity ratio. For this filter, we utilize the total pixels already available for the given component area and the total non-white pixels in the exact location. We calculated the ratio of the same, and if it is between the threshold of 0.25 and 0.60, we considered that component to be valid and added that as part of the final output image. Fig. (8a) shows some elements that are still not identified but are valid parts of the Devanagari word text. Therefore, this character component satisfies the pixel intensity requirements specified above and is included in the final image.

**Fig. 8:**
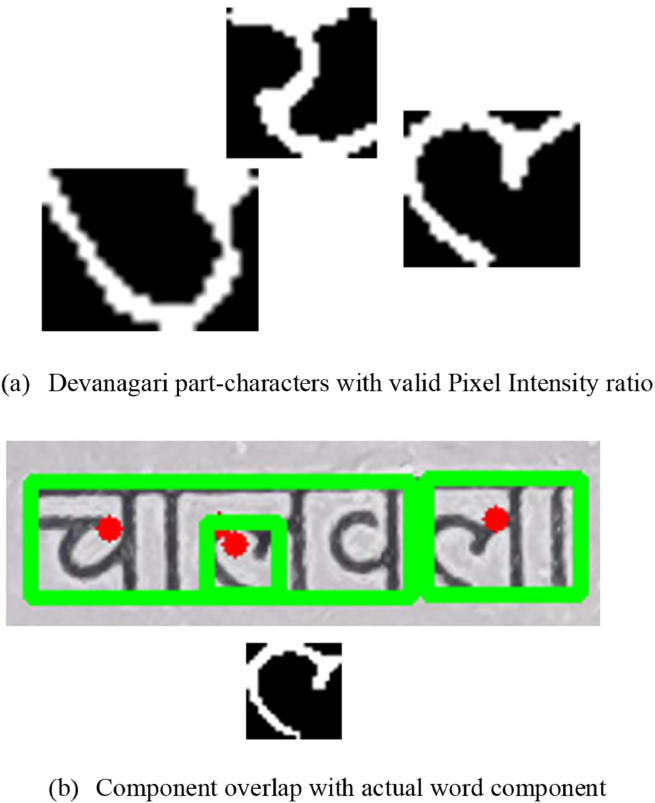
Additional filters detected components.
• Due to the Devanagari characters' tendency to have a lot of curvature, some of the curvature for a given character may be printed separately from the original character. Such components are not treated as part of the original occupying component; hence, an additional filter must be applied for this specific task. To include such components, we used an area-overlapping algorithm for all such components. We calculated the current component's area and the nearest neighbour’s components. If we discover that a component's area overlaps another component by >80 % (OA_P_), we will take that component into account for the final output image. Fig. (8b) depicts one of the components that must be considered because its area completely overlaps with the Devanagari word, which is only a part of itself.

**Fig. 9:**
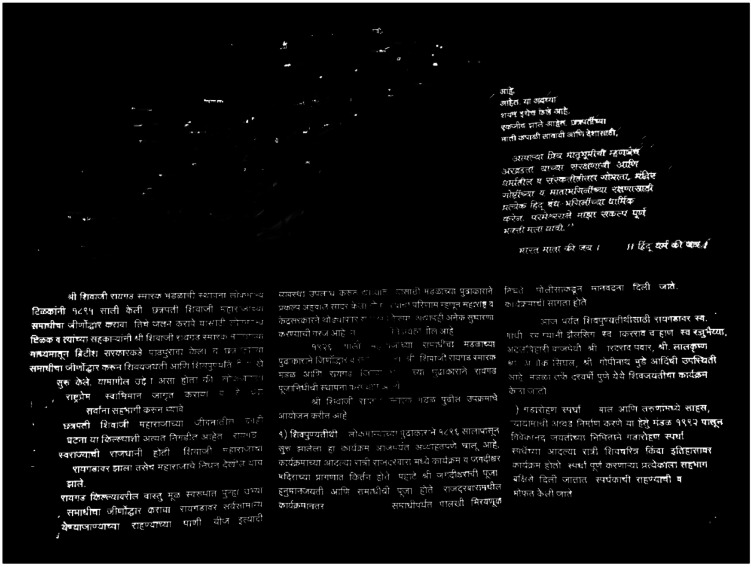
A final image as input to VLM platform.

As a result, by performing extra cluster-level filtering procedures using the Shirorekha and height-based techniques, we can keep many Devanagari text and clusters that have already been detected, leaving only likely Devanagari word text regions in the final image. The methods can remove all non-Devanagari word text components from some images while leaving some non-Devanagari word text components in place for other photos. The accurate Devanagari word text can then be located and identified using VLM platforms Text extraction using the remaining clusters and components. Fig. (9) shows the final image to be used by VLM platforms Text extraction after the application of all the previous clustering and filtering methods. This image contains only the valid components identified by earlier steps. The proposed approach’s computing complexity is O(N^2^). The clustering stage groups connected image components into clusters, and the filtering step applies various filters to the elements in each cluster. The computing order for the clustering step is O(N^2^), where N is set of elements still present after each clustering step. The computational order of the filtering step, where M has become the quantity of elements to which the filtering procedure is utilized in every cluster to O(M).

### VLM platforms

The VLM platforms and their underlying Text extraction algorithms don't just concentrate on accurately recognizing text and characters, but they may also cover:

1. Layout analysis enables them to recognize and understand different elements in an image (such as text, tables, and barcodes).
2. Support for different alphabets, such as English, Greek, and Persian.
3. Support for various input image types, such as TIFF, JPEG, PNG, and PDF, and the ability to export text data across multiple output formats.

With the development of computerized systems, numerous artificial intelligence researchers have attempted to address the issue of VLM platforms Text extraction complexity to develop effective systems that can function accurately and instantly. Although there are numerous VLM platforms with different model versions that are currently available in the market, we will focus on a comparison of five popular VLM platforms: the X Grok 4.1, Microsoft Copilot GPT-5, Google Gemini 3, Bloom AI, ChatGPT 5.2. To activate the Text extraction, each VLM platform was loaded with input image and prompted with “Extract the Devanagari text from the given image”. Keeping the prompt simple and same for all VLM platforms makes the result evaluation and comparison unbiased. The output of the VLMs is compared for original input image and the filtered output image from our proposed method.

## Method validation

The Python 3.12 programming language implements the clustering and filtering methods described in this work. It was executed on an Intel Core 5 (8th generation) CPU with 4 GB of RAM. The Python application required two inputs: a text file with values that determined the cutoff points that would be utilized for the filtration and grouping process and the set of images that needed to be processed for Devanagari text detection. A collection of binary images with the Devanagari text highlighted was the result, which was given as input to different VLM platforms for Text extraction. The processing time for an input image with a resolution of 842 × 595 pixels was 1.2 s. We used images with various versions that we generated ourselves and found online, as described in the earlier information about the data set. A total of 3562 example source images were used to compare the outcomes of our suggested approach. 3223 of these 3562 images were taken from publicly accessible sources, with the remaining images being self-created. We meticulously evaluated the performance of all 5 VLM platforms Text extraction for Devanagari text detection and contrasted their results after applying proposed method across a given data set, instilling confidence in the thoroughness of our evaluation process. The proposed algorithm efficiently reduces the number of components that need to be examined for Devanagari character matching. It successfully identifies Devanagari word texts in the original image from various perspectives, distances, and environmental circumstances, as well as with several typefaces. It can even handle Devanagari word texts with word characters spread across numerous lines. This efficiency means the suggested method can be applied to a wide range of image types, where each line of text may have a variable size or font. By eliminating many non-Devanagari word texts, the suggested method significantly reduces the workload, providing a sense of relief about its efficiency.

**Fig. 10:**
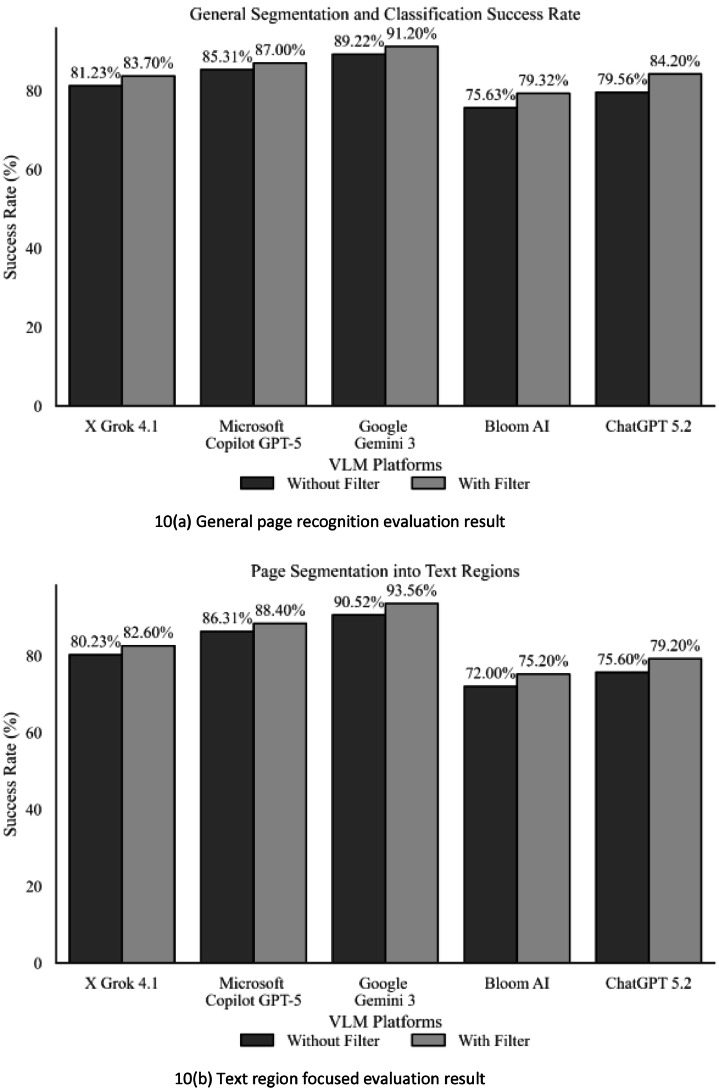
Region representation error rate.

The study [13] recommends evaluating the performance of the proposed method using three quantitative measures. The first evaluation, illustrated in Fig. (10)(a) & (b), assesses the overall page segmentation performance with emphasis on text regions, where errors in non-textual regions are assigned lower weight. The second evaluation focuses on text recognition performance by analyzing the error rates before and after the application of the proposed filter.

The segmentation performance is evaluated using the following error categories:

• Merge: A segmentation output region overlaps with multiple ground truth regions, indicating that distinct ground truth regions have been incorrectly combined into a single detected region.
• Split: Multiple segmentation output regions overlap with a single ground truth region, indicating that a single ground truth region has been incorrectly divided into multiple detected regions.
• Miss / Partial Miss: A segmentation output region fails to fully overlap with the corresponding ground truth region, indicating incomplete or missing detection.
• False Detection: A segmentation output region overlaps with an area where no corresponding ground truth region exists, indicating incorrect detection of a non-existent region.
• Misclassification: A segmentation output region overlaps with a ground truth region but is assigned an incorrect class label.

Table (1) presents the comparative results, demonstrating that the proposed generic filter method significantly reduces the error percentages across all evaluated categories.

**Table 1:** Breakdown of errors made by VLM Platforms before and after filter application.

Relative % Errors	Misclassification		False Detection		Miss/ Partial Miss		Split		Merge	
VLM Platforms	Without Filter	With Filter	Without Filter	With Filter	Without Filter	With Filter	Without Filter	With Filter	Without Filter	With Filter
**X Grok 4.1**	0.23	0.18	0.81	0.76	1.32	1.21	0.81	0.61	1.71	1.51
**Microsoft Copilot GPT-5**	1.62	1.43	0.43	0.39	2.22	1.89	1.71	1.64	2.63	2.35
**Google Gemini 3**	0.13	0.13	0.75	0.67	1.25	1.21	0.83	0.81	1.21	1.12
**Bloom AI**	2.76	2.21	3.54	2.33	5.51	5.25	2.35	2.21	5.21	4.91
**ChatGPT 5.2**	3.42	2.32	2.56	2.01	4.67	3.24	3.12	2.96	3.56	2.12

The following equation provides the term "error rate" according to a conventional definition.

(1) $$WER\;=\frac{\left(iw\;+\;sw\;+\;dw\right)}{nw}$$

**Fig. 11:**
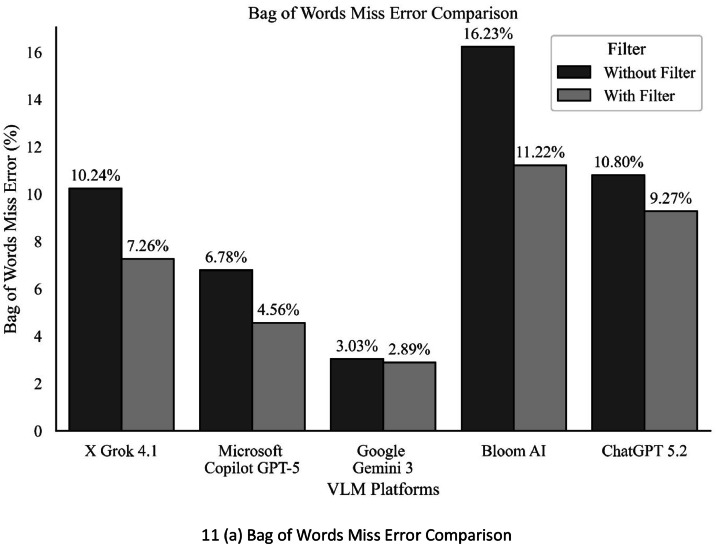

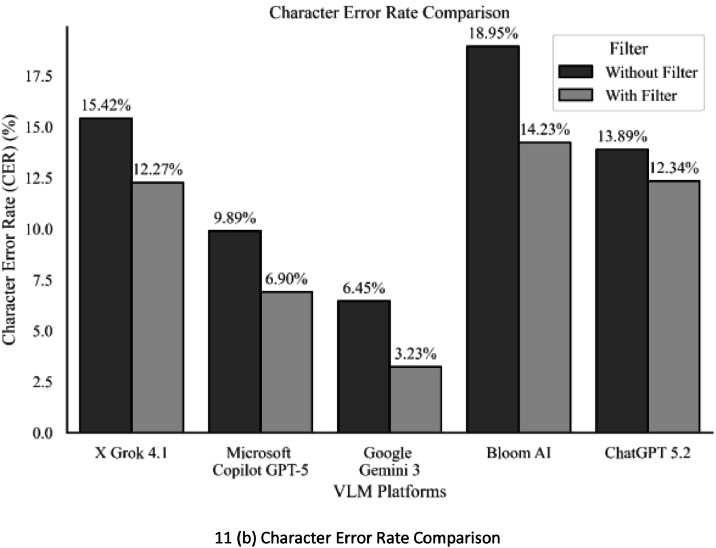
Error rate comparison of VLM platforms.

Our formula is designed with precision. ‘n’ represents a set of images, ‘nw’ denotes the number of words in the source text, ‘sw’ denotes set of words substituted, ‘dw’ denotes set of words eliminated, and ‘iw’ denotes set of words inserted necessary to change the source text’s output into the intended target text. This precision will reassure and instill confidence in the accuracy of the formula. Fig. 11(a) shows the bar chart comparison of the Bag of Word Miss Error Rate for all five VLM platforms before and after applying our Geometrical Filter algorithm on different data sets of mobile captured images. The character error rate is used in a manner like that of:

(2) $$CER\;=\frac{\left(i\;+\;s\;+\;d\;\right)}{n}$$

This utilizes the minimum number of character insertions (i), additions (s), and removals (d) necessary to convert the reference text into the VLM platforms Text extraction output, as well as the set of characters (n). Fig. 11(b) shows a bar chart comparison of the Character Error Rate (CER) for all five VLM platforms before and after the application of our Geometrical Filter algorithm on different data sets of mobile captured images.

Several trials on actual and fictitious data were carried out to validate the correctness, dependability, and performance of the X Grok 4.1, Microsoft Copilot GPT-5, Google Gemini 3, Bloom AI, ChatGPT 5.2. The proposed dataset and ground truth data, such as a list of letters and words present in the Devanagari text images, are included in the experimental setup.

The performance of the proposed model is measured using various evaluation parameters like accuracy, recall, precision, and F-score, as shown below:

(3) $$Accuracy\;=\frac{\left(TP+TN\right)}{\left(TP+TN\;+FP+FN\right)}$$

(4) $$Precision\;=\;\frac{TP}{TP+FP}$$

(5) $$Recall\;=\;\frac{TP}{TP+FN}$$

(6) $$F1\;=\frac{\left(2\;*TP\right)}{2\;*TP+FP+FN}$$

Precision is the ratio of correctly predicted positive classes to the total positive classes. In our research, it is the ratio of correctly predicted types of letters to the proportion of correctly expected in addition to non-predicted types. Recall is the ratio of a correctly predicted positive class to the ratio of all actual classes whether the type is letters or another type. The F1 Score is the weighted average of Precision and Recall. Table (2) Shows the performance result of the different VLM platforms Text extraction evaluation before and after the application of generic filter methods. Table (2) shows that all the VMM platforms produced more promising results on the stated dataset, after the application of Generic filter stages as mentioned previously.

**Table 2:** Result comparison with and without filter application.

	Without Filter					With Filter			
VLM Platforms	Accuracy	Precision	Recall	F1-score	Accuracy		Precision	Recall	F1-score
**X Grok 4.1**	0.775	0.783	0.773	0.775	0.851		0.861	0.862	0.863
**Microsoft** **Copilot GPT-5**	0.86	0.864	0.842	0.854	0.937		0.943	0.935	0.935
**Google** **Gemini 3**	0.913	0.922	0.917	0.912	0.954		0.955	0.953	0.951
**Bloom AI**	0.651	0.675	0.651	0.655	0.802		0.812	0.804	0.814
**ChatGPT 5.2**	0.796	0.82	0.793	0.799	0.871		0.891	0.882	0.886

We have not presumed that the Devanagari text image must have a specific number of characters, sizes, or lines. However, we have employed some threshold values for the grouping and filtering processes. Table (3) lists threshold values that were utilized to show the results in this section. Several representative sample input images from the dataset were considered to arrive at the results. These parameters can be further adjusted for a specific data set to remove more non-Devanagari word text components and find the accurate Devanagari text more rapidly. Alternatively, remove fewer components and get better Devanagari word text identification results. However, for the outcome shown here, we used the identical values stated for each examined data set. Additionally, when testing our data set, we discovered that the flash of mobile cameras frequently resulted in a dark shadow on the obtained photographs, which affected about 32 images. These photographs were manually selected, given different shadow removal pre-processing, and the changed versions were then used as inputs. Like this, approximately 14 photos included unintelligible Devanagari writing, and an output of no text found was deemed successful. Table (2) presents analytical findings from our database’s different image categories to test the accuracy of character extraction by VLM platforms. Table (2) indicates percentage accuracy with and without image enhancement filters.

**Table 3:** Threshold parameter details.

Parameter Name	Threshold Value	Parameter Details
M_P_	3	Minimum pixel size required to keep a component
B_P_	8	Window size for the local thresholding technique in pixels
HW_M_	50	Minimum configurable height and width of CCA components
W_C_	65	Shirorekha width comparison parameter
V_T_	12	Maximum Vertical line count
VH_P_	50	Minimum Vertical height percentage of all Kana
T_P_	25	Min. change percentage on thinning for a probable Devanagari text component
K_N_	4	Maximum neighbour components
HT_D_	5	Maximum percentage height difference
OA_P_	80	Minimum Overlap area of components

### Limitations

This paper proposed an innovative technique to pre-process these images to improve performance, considering the actual VLM platforms Text extraction performance for camera-captured Devanagari document text images. Our method allows for converting these images into binary images with little noise before applying filters one at a time. The experimental findings suggest that VLM platforms accuracy can be improved. The values of and play a crucial role in our program’s pre-processing filters. We are excited about the potential impact of our research and want to investigate how to establish these coefficients more effectively in the future. In this article, we present a versatile approach to detecting Devanagari text, applicable to multiple languages. Our method employs various filtering techniques to identify clustered Devanagari text, without imposing restrictions on the size, quantity, or set text in an image. As demonstrated in Table (2), our approach can accurately identify numerous lines of Devanagari text in an image. This adaptability makes our method less restrictive than most of the previously published work and suitable for various Devanagari-specific languages, font variations, patterns, and situations. The performance of our method was evaluated using benchmark Devanagari text data sets from mobile cameras, and the reported average gains in accuracy of various VLM platforms rates ranged from 85.23 percent to 95.23 percent, as shown in the performance comparison Table (2).

While our proposed approach significantly enhances the identification of Devanagari text, it does have limitations. For instance, if an image is heavily shadowed, cluttered with blots, or blurry, our approach may struggle to extract the components and identify the text accurately if the Devanagari text characters touch the image border.

## Ethics statements

The authors have read and followed the ethical requirements for publication in MethodsX and confirm that the current work does not involve human subjects, animal experiments, or any data collected from social media platforms.

## Related research article

None.

## CRediT authorship contribution statement

**Anup Kelkar:** Validation, Data curation, Writing – original draft, Methodology, Software. **Parag Deshpande:** Conceptualization, Visualization, Software. **O.G. Kakde:** Supervision, Investigation.

## Declaration of competing interest

The authors declare that they have no known competing financial interests or personal relationships that could have appeared to influence the work reported in this paper.

## Data Availability

Data will be made available on request.

## Appendix Supplementary materials

Supplementary material: As a part of validation, a supplement word document is provided which has outputs of some of the VLMs Text Extraction on the sample input images before and after application of our proposed filter method. Image, application 1
