# Supplementary material for: Enhancing camera-captured Devanagari documents via geometric filtering for improved vision-language model text extraction
Source: MethodsX. 2026 May 15;16:103956. doi: 10.1016/j.mex.2026.103956 (PMC13226216; doi:10.1016/j.mex.2026.103956)

**Supplementary Validation:**

1. ChatGPT Text Extraction outputs after loading the original image and passing the prompt as “Extract the Devanagari text from the given image”.


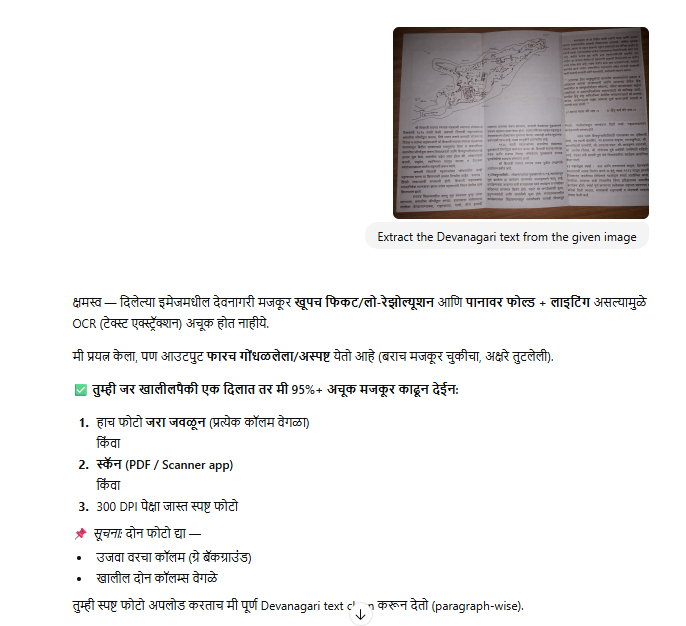


1. Improvement in ChatGPT Text Extraction after loading the filtered image from our proposed algorithm as input. (Prompt is kept same)


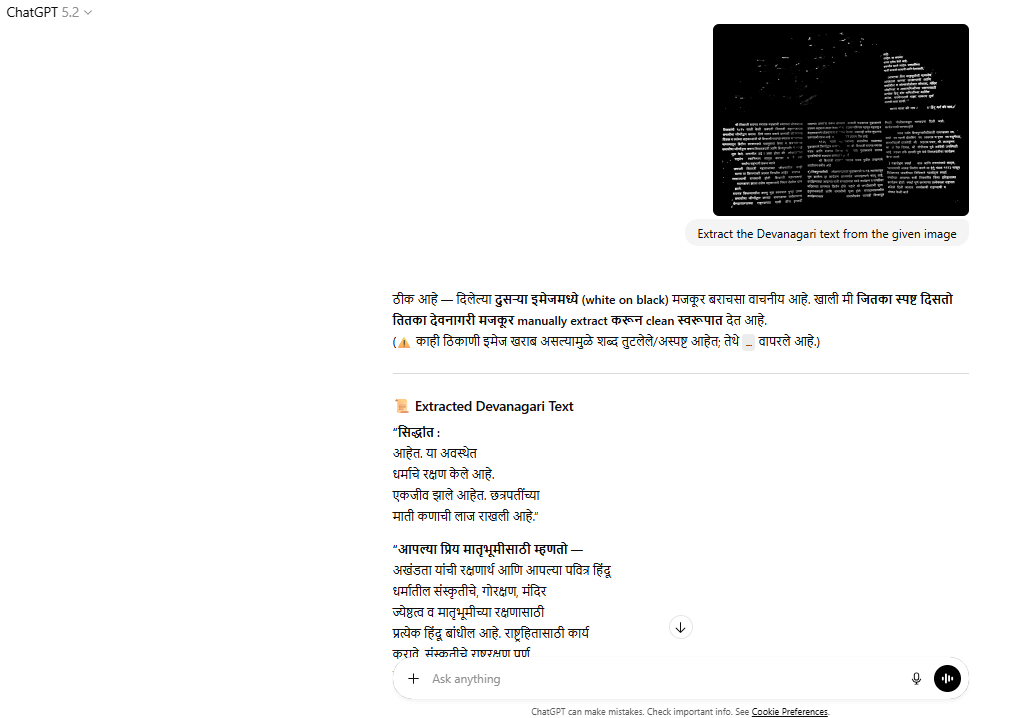


1. Some other VLMs Text Extraction outputs after loading the original image and passing the prompt as “Extract the Devanagari text from the given image”.
2. Windows Copilot output:


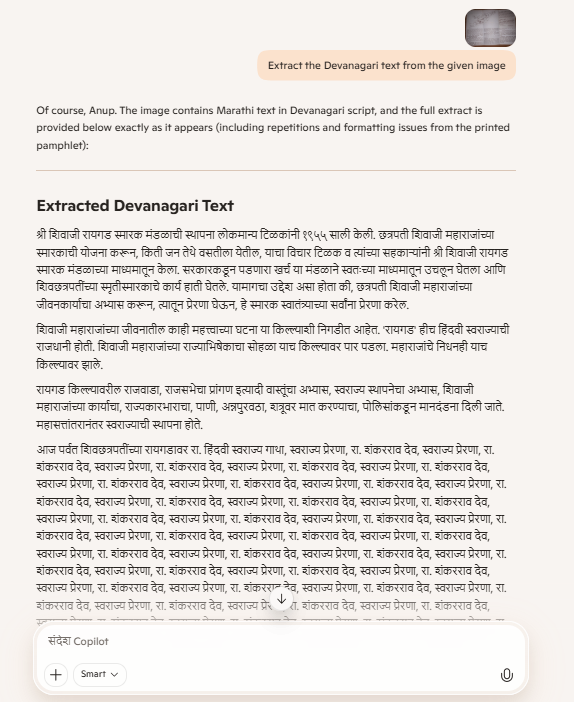


1. Google Gemini output:


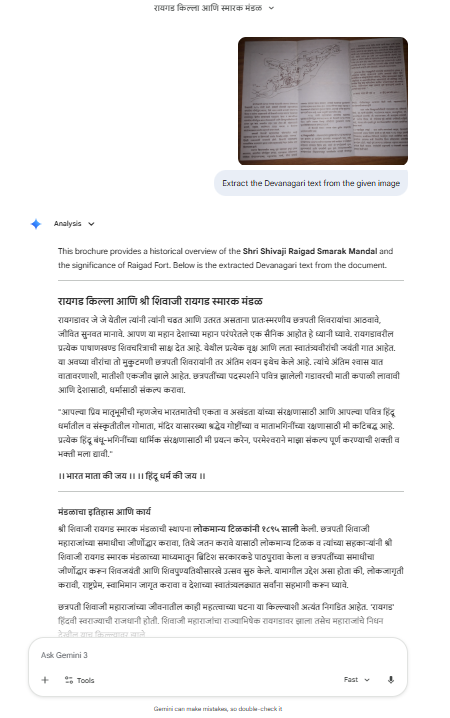


1. Twitter / X Grok output:


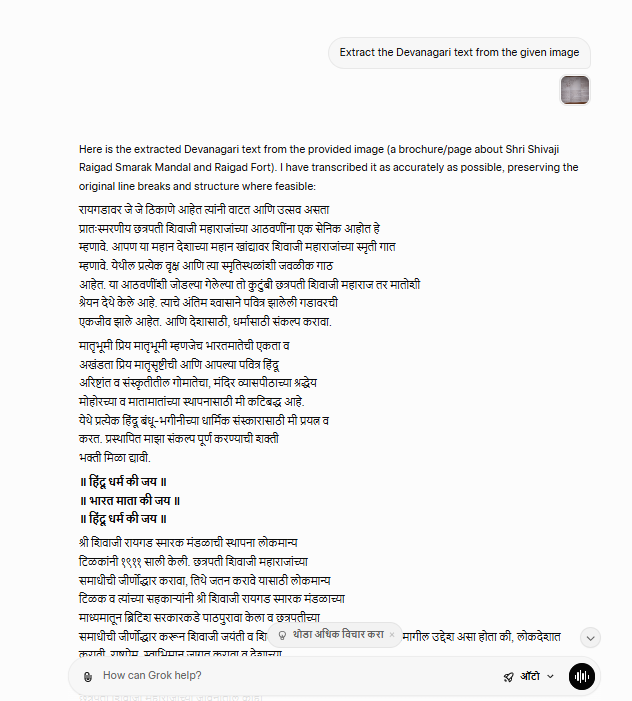

Supplement: Supplementary file 1 — Supplementary material: As a part of validation, a supplement word document is provided which has outputs of some of the VLMs Text Extraction on the sample input images before and after application of our proposed filter method. [file mmc1.docx]
